# Emerging Frontiers in Zebrafish Embryonic and Adult-Derived Cell Lines

**DOI:** 10.3390/ijms26094351

**Published:** 2025-05-03

**Authors:** Álvaro J. Arana, Laura González-Llera, Antón Barreiro-Iglesias, Laura Sánchez

**Affiliations:** 1 Department of Zoology, Genetics and Physical Anthropology, Faculty of Veterinary Science, Universidade de Santiago de Compostela, 27002 Lugo, Spain; 2 Preclinical Animal Models Group, Health Research Institute of Santiago de Compostela (IDIS), 15706 Santiago de Compostela, Spain; 3 Department of Functional Biology, Faculty of Biology, Universidade de Santiago de Compostela, 15782 Santiago de Compostela, Spain; 4 Aquatic One Health Research Center (ARCUS), Universidade de Santiago de Compostela, 15782 Santiago de Compostela, Spain

**Keywords:** zebrafish embryo, cell lines, pluripotency, CRISPR/Cas9, transfection, in vitro models, 3D culture, organoids, toxicology, regenerative medicine

## Abstract

Zebrafish (*Danio rerio*) has become a pivotal vertebrate model in biomedical research, renowned for its genetic similarity to humans, optical transparency, rapid embryonic development, and amenability to experimental manipulation. In recent years, the derivation of cell lines from zebrafish embryos has unlocked new possibilities for in vitro studies across developmental biology, toxicology, disease modeling, and genetic engineering. These embryo-derived cultures offer scalable, reproducible, and ethically favorable alternatives to in vivo approaches, enabling high-throughput screening and mechanistic exploration under defined conditions. This review provides a comprehensive overview of protocols for establishing and maintaining zebrafish embryonic cell lines, emphasizing culture conditions, pluripotency features, transfection strategies, and recent innovations such as genotype-defined mutant lines generated via CRISPR/Cas9 and feeder-free systems. We also highlight emerging applications in oncology, regenerative medicine, and functional genomics, positioning zebrafish cell lines as versatile platforms bridging animal models and next-generation in vitro systems. Its continued optimization holds promise for improved reproducibility, reduced animal use, and expanded translational impact in biomedical research.

## 1. Introduction

Zebrafish (*Danio rerio*) has become a cornerstone of vertebrate biomedical research, renowned for its suitability in developmental biology, genetics, and disease modeling. Key attributes such as external fertilization, optical transparency during early development, small size, and rapid embryogenesis enable real-time imaging and precise experimental manipulation [1,2]. Additionally, the high degree of genetic conservation with humans—approximately 70% genome orthology—underlines its value for translational studies [3]. The ease of embryo collection and cost-effective husbandry further support its widespread use. The introduction of genome editing technologies, particularly CRISPR/Cas9, has significantly expanded the role of zebrafish in functional genomics, enabling precise gene disruption and disease model generation [4].

In parallel with in vivo applications, the establishment of zebrafish cell lines from embryonic and adult tissues has created complementary in vitro systems that support mechanistic studies under defined conditions. Early-stage embryo-derived lines such as ZF4, ZFL, and ZEM2 maintain stable proliferation and exhibit pluripotent or multipotent features across passages [5,6,7]. These cultures provide platforms for toxicological testing, drug screening, and molecular analysis, while also aligning with the ethical principles of the 3Rs—Replacement, Reduction, and Refinement—by reducing reliance on live animal experimentation [8,9]. Their long-term stability and scalability make them increasingly indispensable in vertebrate cellular and molecular biology.

Technological advances have continued to refine these systems. The adoption of feeder-free and chemically defined media has improved reproducibility across laboratories [10,11], while transfection methods, such as nucleofection, have enhanced gene delivery efficiency in zebrafish cells, enabling both transient and stable expression [12]. Optimized CRISPR/Cas9 systems using zebrafish-specific promoters have made in vitro gene editing feasible and reproducible [4]. A recent milestone in the field is the protocol by Geyer et al., which enables the derivation of cell lines from individual 24–36 hpf embryos, coupled with parallel genotyping to generate wild-type and homozygous mutant cultures [13]. This overcomes previous limitations in isolating viable mutant lines from early-lethal or morphologically indistinct phenotypes and facilitates the controlled production of genotype-defined cell lines.

Zebrafish-derived cell cultures have also emerged as powerful tools in oncology. Xenotransplantation of human and murine tumor cells into embryos or clonal zebrafish lines permits the study of tumor growth, metastasis, and host interaction in vivo without the need for immunosuppression [14]. When integrated with 3D culture systems, organoid technology, and transcriptomics, these models enable high-throughput drug screening and mechanistic studies with high resolution and cost-efficiency [15]. Altogether, the integration of zebrafish embryo cell lines with genome editing and advanced in vitro techniques reinforces their utility as versatile platforms for both basic and translational biomedical research.

To construct this review, we conducted a focused literature search using PubMed, Web of Science, and Scopus, emphasizing studies on the generation and biomedical application of zebrafish embryo-derived cell lines. Search terms included “zebrafish embryo cell line”, “zebrafish culture”, “pluripotency zebrafish cell lines”, “zebrafish cell lines transfection”, and “zebrafish organoids”. We prioritized peer-reviewed reports describing original derivation protocols, functional characterization, and innovations relevant to disease modeling, genome editing, and regenerative medicine. Foundational articles were also included to trace the methodological evolution of the field; to further identify and contextualize these primary publications, we additionally consulted Cellosaurus (www.cellosaurus.org, accessed on 28 April 2025), a comprehensive cell line repository. Key information, such as derivation stage, media composition, transfection methods, and lineage potential, was extracted and summarized in comparative tables.

## 2. Overview of Zebrafish Embryo Cell Culture

**Figure 1:**
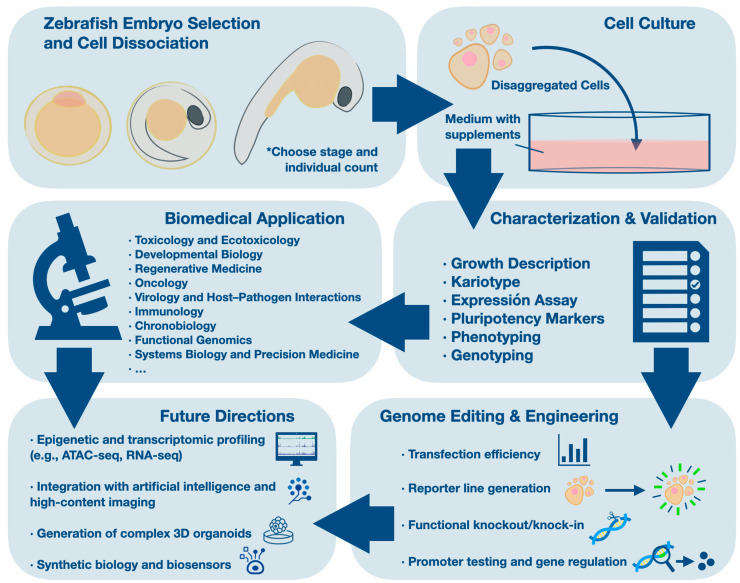
Workflow of zebrafish embryo-derived cell line generation, characterization, and biomedical applications. Schematic overview illustrating the main steps involved in establishing cell lines from zebrafish embryos. Embryos at selected stages are dissociated and cultured under defined conditions to generate stable cell populations. These cultures are subsequently characterized and validated based on growth dynamics, karyotype integrity, gene expression, pluripotency markers, phenotypic traits, and genotyping. Genome editing technologies, including CRISPR/Cas9, enable reporter line generation, functional knockouts, and promoter activity assays. Established cell lines are applied across diverse biomedical fields including toxicology, developmental biology, oncology, immunology, and regenerative medicine. Future directions involve the integration of omics technologies, artificial intelligence, 3D organoid systems, and synthetic biology for enhanced modeling and translational potential.

Zebrafish cell lines are commonly derived from early developmental stages such as the blastula, gastrula, or pre-hatching embryo, when cells exhibit high proliferative capacity and, under appropriate conditions, can retain pluripotent or multipotent features [9]. Early protocols relied on complex media formulations supplemented with fetal bovine serum (FBS), trout serum, or trout embryo extract to support survival and proliferation [1,2,16]. Over time, these media were refined using more defined formulations—such as DMEM, DMEM/F12, and RPMI—often enriched with insulin, selenium, basic fibroblast growth factor (bFGF), and Kit ligand to maintain undifferentiated states and promote sustained growth [8,10]. This general workflow is summarized in Figure 1.

Among available media, Leibovitz’s L-15 has become one of the most widely used for zebrafish and other teleost cell lines. Its buffering capacity without the need for CO_2_ makes it ideal for laboratories without CO_2_ incubators, while its compatibility with culture temperatures of 26–28 °C suits zebrafish biology. Numerous lines, including DRCF, ZFL, PAC2, ZEF1/ZEF2, and PAC2-luc, are maintained in L-15 supplemented with 10–20% FBS, and in the case of ZFL, also with HEPES to stabilize pH [12,15,17,18].

In parallel, alternative media formulations have been developed to meet the specific requirements of distinct cell lines. For example, pluripotent-like lines such as ZES1 and Z428 are maintained in feeder-free DMEM-based media supplemented with bFGF, which support both self-renewal and directed differentiation while eliminating the need for feeder layers [10,11]. Feeder-free systems—defined as culture methods that do not rely on a layer of supportive “feeder” cells—have become essential for improving reproducibility and reducing variability caused by undefined factors secreted by feeder layers. The transition to these systems was also applied to the ZEB2J line, initially established on RTS34st feeder cells and later adapted to feeder-free, serum-based conditions [19]. These lines express canonical stem cell markers—including *nanog*, *sox2*, and *pou5f1*—and have demonstrated the ability to form embryoid bodies and contribute to all germ layers in viv. Other lines such as ZBE3 and PTEN-mutant tumor lines from ptenb−/− or ptena+/− ptenb−/− fish are cultured in standard DMEM with 10% FBS for use in cancer modeling and immune interaction assays [20,21]. ZF4 and ZEB2J are commonly maintained in DMEM/F12. Altogether, the shift toward defined, feeder-free culture conditions enhances reproducibility and facilitates downstream applications such as transfection, gene editing, and directed differentiation.

Beyond media composition, physical and environmental parameters play a crucial role in culture success. Most zebrafish lines are propagated at 26–28 °C under ambient CO_2_. Surface coatings with gelatin or extracellular matrix proteins enhance cell adhesion, and in earlier protocols, feeder layers—particularly RTS34st—were essential for maintaining stem-like phenotypes [22,23]. Some of these feeder cells were genetically engineered to express supportive factors like kit ligand a (*kitlga*) and stromal cell-derived factor 1b (*sdf-1b*), boosting long-term proliferation of primordial germ cells (PGCs) from *vasa::RFP* transgenic embryos [8]. Modern culture strategies now support both self-renewal and directed differentiation into lineages such as neural or muscular cells via specific growth factor combinations [9].

In addition to embryo-derived lines, which are the main focus of this review, several zebrafish cell lines have also been successfully established from adult tissues. This broader landscape reflects the growing interest in developing versatile in vitro platforms beyond early developmental stages. Our primary focus on embryo-derived lines stems from their ethical advantages—since their use aligns with the 3R principles—and from the relative ease of obtaining large numbers of embryonic cells compared to dissociating adult tissues. Moreover, embryonic cells typically exhibit higher proliferative capacity, greater phenotypic homogeneity, and improved amenability to genetic manipulation, making them ideal for scalable and reproducible in vitro studies. Nevertheless, adult-derived lines have demonstrated significant value for specific applications.

Among these, DRCF and SJD.1, derived from adult fin, have been used for transfection studies and environmental toxicology, respectively [17,24,25]. ZF-L, established from adult liver, has become a standard model for hepatotoxicity and xenobiotic metabolism studies, notably expressing inducible *cyp1a1* activity [26]. DrG, from adult kidney, supports cytotoxicity and oxidative stress assays, while ZKS, also from kidney, is used for gene expression studies following electroporation [27,28]. DRM, a muscle-derived line, has been applied to myogenesis research [29].

Of particular relevance are the ZMEL1 melanoma line and *PTEN*-mutant endothelial lines, both derived from adult tumors. ZMEL1, generated from *BRAFV600E*/*p53*−/− melanomas, is widely used for metastasis modeling, drug resistance studies, and CRISPR-mediated genome editing [30], while PTEN-deficient tumor-derived cells enable investigation of tumor angiogenesis and resistance mechanisms in a zebrafish-specific context [21].

Although these adult-derived lines are maintained under culture conditions similar to those used for embryo-derived lines—typically L-15 medium supplemented with 10–20% FBS at 26–28 °C—their applications often address distinct biological questions, focusing on mature tissue physiology, toxicology, and oncology. Nevertheless, comprehensive karyotypic characterization remains limited across both groups, representing a shared constraint when evaluating long-term genomic stability. A comparative overview of zebrafish embryo- and adult-derived cell lines, including their culture characteristics and research applications, is presented in Table 1.

## 3. Protocols for Establishing Cell Lines

The establishment of zebrafish cell lines typically begins with the enzymatic dissociation of early-stage embryos—most commonly at the blastula or gastrula stages—when cells are undifferentiated and highly proliferative. Early efforts to develop zebrafish cell culture systems also explored optimal media formulations, substrate coatings, and culture conditions compatible with embryonic cell proliferation and differentiation [31,32,33]. Treatments with pronase, trypsin, or collagenase enable removal of the chorion and extracellular matrix, yielding single-cell suspensions suitable for seeding onto surfaces pre-coated with gelatin, poly-L-lysine, or extracellular matrix proteins to enhance cell adhesion [2,34]. Early protocols often relied on feeder layers, such as RTS34st cells from rainbow trout spleen, to support cell survival and outgrowth, a strategy used in generating the ZEB2J line [19]. Although these methods initially required pooling 50–100 embryos, current approaches enable culture initiation from single embryos—a significant advance that facilitates genotype-specific studies and reduces animal use. The individualized strategy described by Geyer et al., covered in detail in Section 7, exemplifies this shift [13].

Both haploid and diploid embryos have been used successfully. While haploid cultures are transient and generally unsuitable for long-term maintenance, their hemizygous nature makes them valuable for detecting recessive mutations in screening assays [8,16]. In contrast, diploid-derived lines exhibit greater genomic and proliferative stability, and are typically used in long-term studies across various biological applications.

Several well-characterized lines illustrate the range of derivation strategies. ZEM-2, derived from pooled blastula embryos, was originally cultured in media containing trout extract and serum. Its derivative, ZEM-2A, adapted to a simplified formulation with 5% FBS, shows increased transfection efficiency using viral promoters such as CMV, SV40, and RSV [34]. Similarly, the ZF4 line, derived from 24 hpf embryos, supports stable transfection and prolonged growth [5], while PAC2 cells—also from 24 hpf embryos—have proven useful in circadian biology and CRISPR experiments, particularly in the form of the luciferase-expressing PAC2 reporter line [18,31,33].

Pluripotent-like lines such as ZES1 and Z428 have been derived under feeder-free conditions using DMEM supplemented with bFGF, and are characterized by stable proliferation, embryoid body formation, and expression of stemness markers including *pou5f1* (*oct4*), *nanog*, *sox2*, and *lin28* [10,11]. These lines are particularly suitable for developmental and differentiation studies. Others, such as ZBE3, are optimized for transfection efficiency and viral susceptibility [20], while ZEF1/ZEF2, derived from 5 to 10 somite embryos, support high-efficiency nucleofection for transgene delivery [12].

Beyond embryonic sources, alternative protocols have enabled the derivation of lines from adult tissues and tumor models. The DRCF line, obtained from caudal fin tissue, exhibits fibroblastic morphology and is highly amenable to transfection [17]. Tumor-derived lines, including fibroblast-like PTEN-KO cultures from *ptenb*−/− embryos and endothelial-like cells from spontaneous tumors in *ptena+/−*; *ptenb−/−* fish, provide valuable tools for modeling tumorigenesis, migration, and angiogenesis [21].

These diverse methodologies highlight the versatility of zebrafish cell culture systems, which can be tailored to specific experimental goals. Despite this diversity, a major challenge persists: the lack of standardized benchmarking across cell lines. Comparative data on growth rates, differentiation capacity, or transfection efficiency under equivalent conditions remain scarce. Addressing this gap through systematic studies would enhance reproducibility and help guide the selection of the most appropriate lines for specific assays or screening platforms.

## 4. Pluripotency in Zebrafish Cell Lines

Pluripotency—the capacity of a cell to give rise to all three germ layers—is a defining feature of embryonic stem cells (ESCs) and has been investigated in zebrafish both in vivo and in vitro. Zebrafish ES-like lines such as ZES1 and Z428, derived from blastula-stage embryos under feeder-free conditions, express key transcription factors shared with mammalian ESCs (Table 2) along with alkaline phosphatase (AP) activity as an enzymatic marker [10,11]. These lines can form embryoid bodies and have demonstrated the potential to contribute to all three germ layers in chimera assays, indicating functional pluripotency [22,23].

Despite these similarities, interspecies differences in regulatory programs must be considered. Zebrafish embryonic cells express unique early developmental markers such as *tdgf1* and *gdf3*, which are not conserved in human ESCs [35]. Additionally, while zebrafish ES-like cells express the surface antigen SSEA1 prior to the onset of Pou5f1 and Nanog expression [36], human ESCs are characterized by the expression of markers such as SSEA3, SSEA4, TRA-1-60, TRA-1-81, and CD24, alongside OCT4, SOX2, and NANOG [37]. These distinctions underscore the necessity of species-specific criteria when assessing stemness. A comparative summary of molecular and functional pluripotency features in zebrafish and human ESCs is presented in Table 2.

**Table 2:** Comparative pluripotency features of zebrafish and human ESCs. Molecular markers, functional properties, and species-specific distinctions relevant to stemness evaluation.

Feature	Zebrafish ES-like Lines	Human ESCs	Refs.
Key transcription factors	*pou5f1*, *nanog*, *sox2*, *lin28*	*OCT4*, *SOX2*, *NANOG*	[10,11]
Enzymatic marker	Alkaline phosphatase (AP)	Alkaline phosphatase (AP)	[10,37]
Surface markers	SSEA1	SSEA3, SSEA4, TRA-1-60, TRA-1-81, CD24	[36]
Unique markers (species-specific)	*tdgf1*, *gdf3*	—	[35]
Functional pluripotency	Embryoid body formation; contribution to germ layers	Embryoid bodies; teratoma formation; chimera	[22,23]
Directed differentiation	Neuronal, hepatic, cardiac lineages (e.g., Z428)	Neuronal, cardiac, endodermal, etc.	[7,38]
Pluripotency state	Primed-like	Naïve and primed	[29,39]
Sox2 function	Neural specification	Pluripotency maintenance	[40]
Epigenetic regulation	Less characterized; prone to drift	Defined enhancer usage (*oct4*, *nanog* loci)	[41]

A comparative perspective across model organisms highlights both the potential and current limitations of zebrafish embryo-derived cell lines. In contrast to murine and human pluripotent stem cells, which are extensively characterized using molecular and functional benchmarks such as OCT4, SOX2, and NANOG, zebrafish cell lines still lack standardized criteria for pluripotency. Moreover, while zebrafish blastula-derived cultures can express key genes such as *pou5f1* and *sox2*, the expression of pluripotency-associated proteins in vitro remains low or undetectable under standard conditions [36]. The interpretation of pluripotency in zebrafish must therefore be approached cautiously, and functional assays equivalent to teratoma formation or directed differentiation are still underdeveloped in this species.

Although recent efforts in directed differentiation, transcriptional analysis, and epigenetic characterization have expanded our understanding of zebrafish ES-like cell states, robust functional assays and standardized criteria for assessing pluripotency remain underdeveloped [38,39,40,41]. In comparison, medaka (*Oryzias latipes*) embryonic stem-like cells, such as MES1, retain a diploid karyotype, maintain long-term self-renewal, contribute to chimeras in vivo, and activate regulatory elements from mammalian pluripotency genes [42]. These lines express conserved pluripotency genes including *nanog*, *oct4*, *sall4*, *klf4*, *tcf3*, and *ronin*, and downregulate them upon differentiation, offering a stronger model for understanding vertebrate pluripotency [43].

Other teleosts, such as red sea bream (*Chrysophrys major*), have yielded embryonic stem-like cell lines (SBES1) with in vitro differentiation capacity into multiple lineages including neuron- and muscle-like cells [44]. These comparative examples suggest that the zebrafish model may benefit from further methodological development aimed at stabilizing pluripotent states and validating in vitro differentiation potential.

Additionally, transcriptional network conservation across vertebrates has been documented, but fish-specific differences in marker behavior, such as the early expression of SSEA1 prior to *pou5f1* or *sox2* activation in zebrafish colonies, emphasize the need to establish lineage- and species-specific standards [36,45].

It is important to differentiate between molecular pluripotency, defined by the expression of canonical transcription factors, and functional pluripotency, evidenced by self-renewal and multilineage differentiation in vitro or in vivo. Although zebrafish ES-like lines robustly express key transcription factors, most resemble a “primed” state similar to the post-implantation epiblast, rather than the “naïve” state of the inner cell mass. Regulatory differences support this interpretation: in zebrafish, Sox2 is predominantly linked to neural specification, unlike its central role in pluripotency maintenance in human ESCs [36,40]. Moreover, zebrafish ESCs lack well-characterized epigenetic profiles, such as the differential usage of Oct4 enhancers observed in human systems [43].

Nevertheless, zebrafish lines like Z428 have been directed to differentiate into neuronal, hepatic, and cardiac lineages [7,38], supporting their relevance for regenerative biology and in vitro modeling of lineage specification. These functional capabilities, however, can be compromised by spontaneous differentiation and epigenetic drift, particularly under long-term culture. As such, continuous molecular profiling—via transcriptomic and epigenetic analyses—is critical to validate and preserve the stem-like state during propagation [13]. In sum, zebrafish ES-like lines represent a promising, though still developing, platform for studying vertebrate pluripotency and differentiation.

## 5. Transfection Capabilities and Strategies

The capacity for efficient transfection is a critical parameter when selecting zebrafish cell lines for gene editing, functional assays, or long-term lineage tracing. Early studies demonstrated that lines such as ZEM-2A and ZF4 are amenable to transgene delivery using mammalian promoters—such as CMV, SV40, and RSV—supporting transient expression and, in some cases, stable integration via neomycin resistance [5,34]. PAC2 cells, especially the luciferase-reporter variant, have been widely used in circadian biology, maintaining stable luminescent output for over 20 days, while ZBE3 supports GFP expression and viral infection, making it suitable for host–pathogen interaction studies [18,20].

A broad range of delivery strategies has been applied to zebrafish lines. Chemical transfection agents such as FuGENE HD and Nanofectin have shown moderate success, but certain lines, like ZF4, require physical methods for efficient gene delivery. Nucleofection—an electroporation-based technique—has proven particularly effective in fibroblast-like lines resistant to chemical reagents [12,46]. For example, in ZF4 cells, nucleofection of a pmaxGFP plasmid reached 72% efficiency, compared to only 32% with lipofection using X-tremeGENE HP [46]. Similarly, PAC2 cells showed 40–50% efficiency when nucleofected with GFP plasmids, compared to only 5% using FuGENE 6 [32].

Importantly, delivery systems must be considered in conjunction with the molecular cargos introduced. In addition to fluorescent reporters, functional constructs such as those based on the Tol2 transposon system and CRISPR/Cas9 gene-editing platforms have been tested. Tol2, originally from *Oryzias latipes*, is not a transfection method but a cargo that can be introduced via standard lipofection or electroporation and requires co-delivery with Tol2 transposase mRNA to achieve stable integration. Although commonly used in embryos, successful use in cell lines has been less frequently reported and remains largely qualitative [47,48].

CRISPR/Cas9-based editing has been demonstrated in zebrafish cell lines using plasmid or viral delivery systems. For example, PAC2 cells were successfully edited using a lentiviral vector expressing Cas9 and sgRNA driven by the zebrafish U6 promoter, resulting in functional loss-of-gene expression of *ctgfa*, although editing efficiency was not quantified [4]. In contrast, the DRCF line was transfected with a pEGFP-N1 plasmid using Lipofectamine LTX, confirming transgene expression but without reporting efficiency [17].

These diverse strategies are summarized in Table 3, which compiles delivery methods, cargo types (e.g., GFP, Tol2, and CRISPR), and reported efficiencies across several zebrafish cell lines. While quantitative benchmarks remain limited and sometimes qualitative, the table serves as a practical guide to help researchers align their goals with the most appropriate cell models and transfection protocols.

In some cases, such as PAC2 cells edited with lentiviral CRISPR/Cas9, editing was validated functionally but no quantitative mutation frequency was reported [4]. Future work should emphasize the standardization of transfection protocols across laboratories and the systematic reporting of both transient and stable expression metrics. Optimizing promoter compatibility, delivery conditions, and validation methods tailored to zebrafish cells will be critical for improving reproducibility and expanding their utility in biomedical research.

Zebrafish cell models provide flexibility across applications. Feeder-free lines like ZES1, Z428, and ZBE3 are suited to developmental biology and viral infection models [10,11,20], while ZF4, ZEM-2A, and PAC2-luc are optimal for high-throughput reporter assays and pharmacological screens [18,34]. However, standardized quantitative benchmarks for transfection efficiency across zebrafish lines remain scarce. Table 3 offers a consolidated starting point for guiding experimental design and evaluating methodological feasibility.

## 6. Applications of Zebrafish Cell Lines

Zebrafish cell lines, derived from both embryonic and adult tissues, have become indispensable tools in biomedical and environmental sciences. Their vertebrate origin, high genetic similarity to humans, and responsiveness to genetic and pharmacological manipulation make them highly suitable for diverse in vitro applications. These include toxicology, developmental biology, oncology, immunology, neurobiology, chronobiology, and increasingly, regenerative and translational medicine. Serving as simplified yet biologically relevant systems, these cultures complement in vivo zebrafish models by offering scalable platforms for controlled, high-throughput experimentation.

In toxicological research, several zebrafish lines have demonstrated strong utility in environmental monitoring and xenobiotic metabolism studies. The ZFL line, derived from adult liver tissue, is a benchmark model for hepatic toxicity, particularly through inducible expression of cytochrome P450 enzymes like *cyp1a1* in response to TCDD [6,26]. ZF4 cells, obtained from 24 hpf embryos, are widely used to assess the toxicity of nanoparticles, pesticides, and endocrine-disrupting compounds [15,32]. Meanwhile, the ZEM2S line, adapted to simplified serum-free media, has been applied in ecotoxicological testing, supporting reproducible and ethically sound assays [32].

In developmental biology, ES-like lines such as ZES1 and Z428 offer robust platforms for studying early lineage specification and stemness. These lines express canonical pluripotency markers, form embryoid bodies in vitro, and contribute to germ layers in chimeric assays [10,11,22,23]. They can be directed toward specific lineages—including neuronal, hepatic, and cardiac fates—under defined protocols, thus providing valuable models for vertebrate development and regenerative research [34,38]. Their functional analogy to mammalian ESCs makes them key models for exploring evolutionarily conserved pathways.

In the oncology field, zebrafish cell lines have enabled both in vitro and in vivo cancer studies. Tumor-derived lines from *ptena+/−*; *ptenb−/−* backgrounds recapitulate hallmarks of cancer such as angiogenesis, migration, and drug resistance [21]. These cells support detailed analysis of tumor biology and integrate well with xenotransplantation assays in zebrafish embryos. The development of zebrafish patient-derived xenograft (zPDX) models further enhances their translational value, offering a cost-effective, vertebrate-compatible platform for drug screening and personalized therapy evaluation [49]. Coupled with transcriptomic and imaging techniques, these models allow real-time monitoring of tumor progression and treatment response.

Zebrafish lines also support virology, immunology, and chronobiology studies. For instance, ZBE3 is susceptible to viral infection and supports GFP expression, facilitating real-time analysis of viral replication and host–pathogen interactions [20]. PAC2 and ZEB2J have been used to study innate immune pathways and stress responses, while the PAC2-luc line enables monitoring of circadian gene expression through its stable luciferase reporter system [18].

Recent advances in molecular tools have broadened the experimental scope of zebrafish cell cultures. ZEF1 and ZEF2 fibroblasts from somite-stage embryos, as well as the DRCF line from adult fin, show high compatibility with nucleofection and transgene expression [12,17]. The implementation of CRISPR/Cas9 editing directly in vitro now enables the generation of mutant cell lines from individually genotyped embryos, streamlining functional genomics and reducing reliance on animal experimentation [13].

From a translational perspective, zebrafish cell lines are bridging basic research and clinical innovation. Their conserved signaling networks and drug metabolism profiles support the modeling of human disease variants and compound screening in scalable, ethically favorable systems. However, direct comparison with mammalian models remains limited. Systematic benchmarking of drug metabolism, genome editing fidelity, and stress response dynamics would help define their role in preclinical pipelines and identify context-specific advantages. Future efforts focused on standardizing culture conditions, validating long-term pluripotency, and integrating omics approaches will be crucial to position zebrafish cell lines as robust, reproducible, and versatile tools for personalized medicine, toxicology, and regenerative biology.

## 7. Generation of Genotype-Defined Zebrafish Cell Lines from Individual Embryos

The establishment of zebrafish cell lines from individually genotyped embryos marks a significant methodological advance in functional genomics. Conventional protocols typically involve pooling dozens or hundreds of embryos from incrosses to ensure sufficient cell yield and promote outgrowth. While effective for wild-type or homogeneous populations, this approach poses limitations when studying early-lethal mutations or phenotypes without overt morphological features, where homozygous mutants cannot be distinguished visually. As a result, generating stable mutant cell lines from such backgrounds has remained a longstanding challenge, restricting in vitro analysis of numerous developmental and disease-related genes.

To address this, Geyer et al. introduced a robust and scalable method for deriving zebrafish cell lines from individual 24–36 hpf embryos [13]. The procedure begins with dechorionation and bleaching of each embryo to reduce microbial contamination. Enzymatic dissociation using pronase and trypsin yields a single-cell suspension, which is seeded into individual wells of a 96-well plate. In parallel, an aliquot of dissociated cells is processed for PCR-based genotyping. This dual workflow enables retrospective identification of the genotype—wild-type, heterozygous, or homozygous mutant—associated with each well, circumventing the need for morphological selection and allowing precise assignment of genetic identity to each culture.

Following genotyping, wells containing cells of the desired genotype can be expanded and, if needed, pooled to establish clonal or monoclonal lines. While not all wells reach confluence, the protocol reports high success rates, with up to 80% of uncontaminated cultures achieving sufficient outgrowth. Crucially, embryos are collected after zygotic genome activation, ensuring transcriptional competence and proliferative capacity in derived cells. This method is particularly advantageous for studying early-lethal mutations or genotypes incompatible with adult viability, offering a direct route to mutant cell lines without the need to maintain or breed homozygous adult fish.

The protocol also provides important ethical and logistical benefits. By eliminating the need to rear embryos to feeding stages or adulthood, it aligns with the 3Rs reducing animal use and potential suffering. Moreover, because the entire procedure occurs before the onset of autonomous feeding, it falls outside the scope of animal experimentation under EU Directive 2010/63/EU, streamlining regulatory requirements while maintaining high ethical standards.

Cell lines derived from single, genotyped embryos are highly suitable for downstream applications such as transcriptomics, genome editing, compound screening, and phenotypic characterization under defined in vitro conditions. Their genetic uniformity enhances reproducibility and facilitates the direct linkage between genotype and cellular phenotype.

Additional strategies have also been explored to generate genotype-defined zebrafish cell cultures. For instance, Venta et al. described the establishment of cell lines derived from gynogenetic diploid embryos, providing genetically homogeneous cell populations without paternal DNA contribution [50]. This approach not only facilitates the derivation of lines with simplified genetic backgrounds but also offers a complementary method to single-embryo genotyping for studying the cellular consequences of specific maternal or recessive mutations.

In summary, the single-embryo derivation protocol developed by Geyer et al. opens new avenues for generating genetically defined zebrafish cell lines [13]. It overcomes previous technical and ethical limitations, enabling high-resolution, reproducible studies of mutant phenotypes in a vertebrate system and reinforcing the zebrafish model’s role in scalable, genotype-driven biomedical research.

## 8. Efficiency and Challenges

The efficiency of establishing and maintaining zebrafish cell lines depends on multiple variables, including the embryonic stage of isolation, enzymatic dissociation methods, medium composition, use of feeder layers, and the capacity of the culture system to preserve both genetic and phenotypic stability over time. Early protocols, such as those described by Collodi et al., relied on pooling blastula-stage embryos and employing complex media supplemented with trout serum and embryo extract [2]. Although these methods produced lines such as ZEM-2 and ZEM-2A, they often lacked reproducibility and scalability.

Subsequent advances—including the use of chemically defined, feeder-free media enriched with fish serum and growth factors like bFGF—have markedly improved culture stability and proliferative capacity. This is exemplified by the successful establishment of ES-like lines such as ZES1 and Z428, which express key pluripotency markers and are maintained over extended passages [10,11]. Despite these improvements, several limitations remain that hinder the widespread and standardized use of zebrafish cell lines.

One major concern is genomic instability. Many widely used lines exhibit abnormal karyotypes (Table 1). For instance, ZF4 is hyperploid, with an average chromosome count of ~120 and ZEM-2A shows a modal number of 77 [5,34]. Such chromosomal abnormalities can alter gene expression profiles, cellular behavior, and response to experimental manipulations. Genomic instability in zebrafish cell lines has been directly assessed in some cases. For example, He et al. demonstrated by flow cytometry analysis that PAC2 and ZF4 cells display abnormal DNA content and polyploidy, supporting the need for routine cytogenetic validation [32]. These findings underscore the importance of implementing standardized chromosomal monitoring protocols to maintain data reliability. This variability poses a significant barrier to reproducibility and highlights the need for routine cytogenetic validation. Without standardized karyotyping or genomic authentication, cross-study comparisons and translational applications may be compromised.

A critical future direction is the implementation of rigorous quality control frameworks for zebrafish cell lines. Unlike mammalian cell culture, where repositories such as European Collection of Authenticated Cell Cultures (ECACC, https://www.culturecollections.org.uk/ECACC, accessed on 5 April 2025) or American Type Culture Collection (ATCC, https://www.atcc.org/, accessed on 5 April 2025) require thorough authentication, most zebrafish lines are maintained without formalized validation. This increases the risk of misidentification, contamination, or genetic drift over time. Based on current zebrafish cell culture literature, we recommend that quality assessment should minimally include: (i) regular mycoplasma testing, (ii) karyotype analysis to monitor chromosomal integrity, (iii) phenotypic stability assessments based on morphology and growth rate monitoring, and (iv) genetic identity verification, ideally through zebrafish-specific short tandem repeat (STR) panels or targeted gene-specific PCR assays.

To facilitate standardization, we propose a practical checklist of essential quality controls that can be adopted by laboratories working with zebrafish cell lines (Table 4). This framework is grounded in current zebrafish practices, including mycoplasma testing and genetic validation, karyotyping and phenotypic monitoring, and emerging STR-based authentication strategies [13,32,50].

Venta et al. developed a zebrafish-specific 13-plex STR panel comprising tetra- and pentanucleotide repeats, designed for the authentication of individual zebrafish and laboratory strains with high accuracy [50]. This tool can be readily adapted to verify the genetic identity of zebrafish-derived cell lines, providing an additional safeguard against contamination, mislabeling, and genetic drift. Although STR profiling is not yet routinely applied in zebrafish in vitro systems, its adoption would align with best practices established in mammalian cell culture and substantially improve quality assurance and inter-laboratory reproducibility.

Maintaining pluripotency in ES-like lines also remains challenging. Although ZES1 and Z428 exhibit stable expression of canonical transcription factors such as *pou5f1*, *nanog*, and *sox2*, spontaneous differentiation frequently occurs, even under optimized feeder-free and serum-reduced conditions [10,11]. This may reflect intrinsic differences in signaling dynamics and chromatin regulation between zebrafish and mammals, or suboptimal adaptation of mammalian ESC culture protocols to teleost systems. While chemically defined media have improved consistency, the field still lacks a zebrafish-specific pluripotency maintenance system analogous to those developed for mouse or human ESCs. Moreover, the absence of feeder cells—though beneficial for ethical and technical reasons—may accelerate loss of stem-like features over time, necessitating regular monitoring of marker expression and differentiation potential.

Transfection efficiency is another technical bottleneck. While lines like ZEM-2A and PAC2 respond well to chemical reagents such as FuGENE HD and Nanofectin [18,34], others like ZF4 are refractory to chemical transfection and require electroporation-based methods such as nucleofection [12]. Variability in transfection outcomes likely arises from differences in membrane composition, chromatin accessibility, or cell cycle status. Moreover, the limited availability of zebrafish-optimized promoters, selection markers, and expression vectors restricts the generalizability of transgenic and gene-editing protocols. Broader access to molecular tools tailored to zebrafish biology would enhance reproducibility and facilitate standardization across laboratories.

Despite these limitations, zebrafish cell lines remain powerful platforms for in vitro research. They offer scalable, cost-effective, and ethically favorable alternatives to animal models in areas such as high-throughput toxicity testing, genetic manipulation, and disease modeling. To fully realize their potential in biomedical research, however, rigorous standardization and quality control are imperative. This includes genomic authentication (e.g., karyotyping, STR profiling), regular mycoplasma testing, documentation of passage number, and functional characterization of stemness and differentiation capacity. The establishment of centralized zebrafish cell line repositories—similar to ATCC or ECACC—would further support reproducibility, reduce redundancy, and promote cross-laboratory consistency.

While such technical bottlenecks persist, adherence to routine quality control practices (Table 4) would mitigate variability and enhance data reliability across studies. Integrating these checks into standard workflows is a crucial step toward improving reproducibility and enabling broader adoption of zebrafish in vitro models in translational research. As zebrafish cell culture technologies continue to evolve, addressing these technical and standardization challenges will be essential to strengthen their role in modern cell biology, functional genomics, and translational applications.

## 9. Cost-Effectiveness and Comparative Utility of Zebrafish Embryonic Cell Lines vs. Live Embryo Assays

Although zebrafish embryonic cell lines represent a powerful tool for mechanistic in vitro studies, their establishment and maintenance require considerable initial investment in terms of time, technical expertise, and resources. Deriving these lines involves enzymatic dissociation of blastomeres, the use of enriched media containing supplements such as bFGF and embryo extract, and long-term culture under tightly controlled conditions [9,10]. However, once stabilized, these cell lines can be maintained indefinitely, cryopreserved, and used in high-throughput screening (HTS) platforms, such as 96- or 384-well plates, significantly reducing per-experiment costs over time [51]. Their scalability, combined with faster assay turnaround times and straightforward readouts for endpoints such as mitochondrial function or membrane integrity, makes them highly suitable for cytotoxicity and early-stage toxicity assays, particularly in HTS applications [48,51].

In contrast, live zebrafish embryo cultures are cost-effective at the initial stages, requiring only basic equipment and minimal reagents. Zebrafish produce large numbers of externally developing embryos that are easy to manipulate and compatible with automated imaging and analysis pipelines [52]. Embryo-based assays, such as the Fish Embryo Toxicity Test (FET), are well established and offer a whole-organism context, which remains critical for studying developmental processes, systemic responses, and teratogenic effects [25,53,54]. Nonetheless, the manual handling of embryos and the longer observation periods required—often several days—can limit throughput and introduce variability between replicates and laboratories [55].

Zebrafish cell lines offer a high degree of reproducibility due to their genetic homogeneity and standardized culture conditions, making them well suited for interlaboratory comparison and regulatory applications [48,51]. In contrast, live embryo tests—although standardized and validated—are subject to greater variability due to biological and environmental factors [55]. Moreover, both systems align with the 3Rs principle, but embryonic cell lines provide an additional ethical advantage by eliminating the need for live animals in repeated assays, especially when derived from embryos prior to the onset of independent feeding [9,55].

Taken together, zebrafish embryonic cell lines and live embryo cultures each offer distinct advantages depending on the experimental goal. Cell lines are particularly advantageous for high-throughput screening, mechanistic assays, and long-term studies requiring reproducibility and scalability. Live embryo cultures, meanwhile, remain indispensable for in vivo validation, developmental biology, and regulatory toxicology due to their physiological complexity and predictive power at the organismal level [25,52,54]. A comparative overview of both systems is presented in Table 5.

## 10. Future Perspectives

The continued evolution of zebrafish cell culture hinges on refining existing methodologies while integrating innovative technologies to enhance reproducibility, biological relevance, and translational potential. One of the most immediate priorities is the development of chemically defined, xeno-free media tailored specifically to zebrafish cells. Current reliance on FBS, fish serum, or trout embryo extract introduces batch variability, limits standardization, and raises ethical concerns. Replacing these undefined components with recombinant growth factors and fully defined supplements would promote inter-laboratory reproducibility and align zebrafish systems with regulatory and clinical standards [10,11]. Such formulations could also improve the maintenance of pluripotency and enable precise, lineage-directed differentiation under controlled conditions—key requirements for modeling development and disease in vitro.

A promising direction involves the expansion of three-dimensional (3D) culture models, including spheroids and organoids. Zebrafish liver and embryonic cells have already been adapted to 3D formats using hanging-drop systems, hydrogel matrices, and low-adhesion platforms [15]. These models recapitulate tissue architecture more faithfully than 2D cultures, offering enhanced cellular organization, more physiologically relevant gene expression, and greater utility in toxicology, oncology, and pharmacological testing. The incorporation of co-culture systems—either with different zebrafish lineages or with mammalian cells—adds another layer of complexity, enabling in vitro modeling of intercellular communication, immune interactions, and tumor–stroma dynamics.

Recent advances demonstrate the feasibility of adapting 3D culture techniques and organoid models to zebrafish-derived cell lines. Park et al. reported that zebrafish liver and embryonic cell lines such as ZFL and ZEM2S can be cultured under low-attachment conditions using DMEM/F12 supplemented with 10% FBS, leading to the spontaneous formation of uniform spheroids without additional scaffolds [56]. This three-dimensional (3D) spheroid-based in vitro platform showed enhanced viability, stable morphology, and increased expression of differentiation-associated markers compared to traditional 2D cultures. Furthermore, hepatic organoids derived from ZFL cells under these conditions displayed apical microvilli and polarized cellular organization, closely resembling in vivo liver architecture.

Despite these promising results, the field remains underdeveloped compared to mammalian organoid systems. Standardized protocols for zebrafish spheroid formation, matrix embedding, and long-term maintenance are lacking, and comparative transcriptomic or functional validation are still rare. Nevertheless, the successful adaptation of organoid technology to zebrafish cell lines offers an unprecedented opportunity to study cell–cell and cell–matrix interactions, tissue-specific differentiation, and environmental toxicity in a physiologically relevant context. Looking ahead, the development of advanced zebrafish in vitro models will likely involve the incorporation of three-dimensional (3D) culture systems, organoid technologies, and co-culture platforms. These systems offer enhanced physiological relevance and can better replicate in vivo architecture and signaling gradients. Although 3D culture is well established in mammalian systems, only limited efforts have been made to adapt these strategies to zebrafish-derived cell lines.

Furthermore, integrating transcriptomic and epigenomic profiling (e.g., RNA-seq, ATAC-seq) will be crucial for defining cellular states and regulatory programs. Although RNA-seq has been widely applied to zebrafish-derived cell lines, chromatin accessibility profiling using ATAC-seq remains largely unexplored in these in vitro systems. Recent studies have successfully implemented ATAC-seq in zebrafish embryos, as well as in sorted in vivo cell types such as neurons and osteoblasts, providing valuable insight into gene regulatory elements during development [57,58,59]. However, no studies to date have applied ATAC-seq to zebrafish cell lines such as ZF4, ZFL, or PAC2. This lack of epigenomic data in cell lines represents a major knowledge gap—and an opportunity to explore chromatin remodeling, lineage specification, and responses to environmental stimuli in vitro.

Emerging technologies in synthetic biology, biosensor design, and artificial intelligence (AI) promise to transform zebrafish cell lines into powerful platforms for systems biology and precision medicine. Engineered zebrafish cells carrying synthetic gene circuits could serve as living biosensors for environmental toxins or therapeutic compounds. Simultaneously, AI-driven analysis of high-content imaging and multi-omics data may allow predictive modeling of cellular behaviors, drug responses, or disease states. These advances will depend on expanding the toolkit of zebrafish-specific molecular resources—including inducible promoters, CRISPRa/i systems, and optogenetic modules for spatiotemporal control of gene expression.

The recent development of protocols for deriving cell lines from individually genotyped embryos represents a paradigm shift in functional screening and disease modeling. These monoclonal mutant lines facilitate the study of early-lethal mutations, genotype-specific drug responses, and cell-intrinsic transcriptional phenotypes without requiring large-scale breeding [13]. When combined with CRISPR-based genome editing and single-cell transcriptomics, such systems offer high-resolution interrogation of gene function, epistatic interactions, and regulatory pathways in a vertebrate model.

Importantly, zebrafish cell cultures align closely with the ethical principles of the 3Rs (Replacement, Reduction, and Refinement). Their derivation from early-stage embryos, compatibility with multiwell formats, and scalability for high-throughput assays reduce the need for live animal experimentation. As global regulatory frameworks increasingly encourage non-mammalian alternatives for toxicity and efficacy testing, zebrafish-derived lines may fill a critical niche between mammalian cell cultures and whole-organism models.

Recent methodological advances, including the generation of cell lines from individually genotyped embryos and the introduction of zebrafish-specific STR panels for genetic authentication, further enhance the precision and reproducibility of zebrafish in vitro models. However, broader adoption of quality control standards—such as STR profiling, regular karyotyping, and molecular validation—remains essential to fully realize their translational potential.

In conclusion, zebrafish cell lines are rapidly transitioning from experimental platforms to foundational tools in developmental biology, toxicology, oncology, and regenerative medicine. With ongoing progress in culture standardization, genomic fidelity, quality control, and functional validation, these models are poised to play a central role in modern biomedical research. Their unique combination of vertebrate-specific features, genetic accessibility, scalability, and ethical compatibility makes them ideal candidates for next-generation in vitro systems bridging basic science and translational innovation.

## Figures and Tables

**Table 1:** Summary of zebrafish cell lines derived from embryos and adult tissues, including culture characteristics and research applications.

Embryo-Derived Cell Lines							
Cell Line	Source	Embryos Used	Culture Medium	Karyotype	Transfection	Application	Refs.
PAC2	24 hpf embryos	~50 embryos per culture	L-15 + 15% FBS	NR	FuGENE HD, Nanofectin; luciferase stable	Circadian and CRISPR studies; light-entrainable reporter	[18,31,32,33]
PAC-2 luc reporter	Transfected PAC-2	24 hpf; number not specified	L-15 + 15% FBS + G-418 (stable clones)	NR	Luciferase (stable > 20 d)	Circadian studies; light-inducible gene expression	[18]
PTEN-KO	Single ptenb−/− embryo	1	DMEM + 10% FBS	NR	Lipofectamine	Tumor and migration modeling	[21]
Z428	Blastula-stage embryos	200	DMEM + 10% FBS + bFGF (feeder-free)	Diploid (48 chr)	High efficiency; stable GFP	Pluripotency; 3 germ layers	[11]
ZBE3	Blastula-stage embryos	Not specified	DMEM + 10% FBS	NR	High efficiency	Virus–host interactions; immune studies	[20]
ZEB2J	Blastula-stage; from ZEB2	Not specified	DMEM/F12 + 15% FBS (initially with RTS34st)	Heteroploid	NR	ES-like; adherent; Pou-2 expression	[19]
ZEF1/ZEF2	5–10 somite embryos	Not specified	L-15 + 10% FBS + antibiotics	NR	Nucleofection (up to 43% GFP+)	Fibroblast cultures; high nucleofection	[12]
ZEM-2	Blastula-stage embryos	10 embryos/well (batch)	Trout extract + FBS (complex medium)	Aneuploid (modal = 73)	CMV, SV40, RSV active	Neural and early development studies	[2,7]
ZEM-2A	Derived from ZEM-2	Derived from ZEM-2	5% FBS (simplified medium)	Aneuploid (modal = 77)	CMV, SV40, RSV active	Transfection studies; simplified conditions	[34]
ZEM2S	Selected from ZEM-2	Derived from ZEM-2	DMEM/L-15 (no trout extract)	NR	NR	Cytotoxicity; ecotoxicology	[15,32,33]
ZES1	Blastula-stage embryos	200	DMEM + 10% FBS + bFGF (feeder-free)	Diploid; stable	FuGENE HD; stable GFP	Pluripotency; multi-lineage differentiation	[10]
ZF4	24 hpf embryos	50 per batch	DMEM/F12 + 10% FBS	Hyperploid (~120)	Efficient; supports CMV, RSV, SV40	Gene regulation; wound healing; cancer research	[5,32]
**Adult-Derived Cell Line**							
**Cell Line**	**Source**	**Culture Medium**	**Karyotype**	**Transfection**	**Application**	**Refs.**	
DRCF	Adult fin-derived	L-15 + 20% FBS	Modal diploid = 50 (range of 32–60)	Lipofectamine LTX + Plus Reagent	Transfection studies; fibroblastic morphology	[17]	
SJD.1	Adult caudal fin	L-15 + 10% FBS	NR	Lipofectamine 2000	Environmental toxicology	[24]	
ZF-L	Adult liver	L-15 + 15% FBS + HEPES	NR	Lipofectamine 2000	Cytotoxicity; ecotoxicology/Hepatotoxicity studies	[15,26]	
DrG	Adult kidney	L-15 + 10% FBS, 28 °C	NR	Lipofectamine 2000	Cytotoxicity assays, oxidative stress studies	[27]	
ZKS	Adult kidney	L-15 + 10% FBS, 28 °C	NR	Electroporation	Gene expression studies	[28]	
DRM	Adult muscle	L-15 + 20% FBS, 28 °C	NR	NR	Myogenesis studies	[29]	
PTEN-mutant tumor	ptena+/− ptenb−/− tumor (adult)	L-15 + supplements; room temp	NR	NR	Endothelial markers; tumor and vascular studies	[21]	
ZMEL1	Melanoma in adult	DMEM + 10% FBS, 28 °C	NR	Nucleofection	Metastasis modeling, CRISPR	[30]	

NR—Not reported in the original publication.

**Table 3:** Reported transfection and gene editing efficiencies in zebrafish cell lines.

Method	Cell Line	Cargo	Efficiency Type	Efficiency (%)	Refs.
Nucleofection	ZF4	pmaxGFP plasmid	Transfection	50–75	[46]
Nucleofection	PAC2	pmaxGFP plasmid	Transfection	40–50	[32]
Lipofection	ZF4	pmaxGFP plasmid	Transfection	3–5	[46]
Lipofection	PAC2	GFP plasmid	Transfection	Moderate (qual.)	[31]
Lipofection	PAC2	Tol2 construct + transposase	Transfection	Stable integration (qual.)	[47,48]
Lipofection	DRCF	pEGFP-N1	Transfection	Not reported (GFP positive)	[17]
Lentivirus	PAC2	Cas9 + sgRNA vector	Editing	Not reported (functional knockout confirmed)	[4]
FuGENE 6	ZF4	GFP plasmid	Transfection	15–20	[32]
FuGENE 6	PAC2	GFP plasmid	Transfection	~5	[32]

**Table 4:** Recommended quality control measures and monitoring frequency for zebrafish cell lines.

Control Parameter	Method	Frequency	Refs.
Mycoplasma contamination	PCR-based or luminescent assay	Monthly	[13]
Chromosomal stability	Karyotype analysis (Giemsa staining or DAPI) or nuclear DNA content by flow cytometry	Every 10 passages	[32]
Genetic identity	Zebrafish-specific STR profiling or gene-specific PCR validation	At establishment and yearly	[13,43]
Phenotypic consistency	Morphology assessment and doubling time monitoring	Each passage	[32]
Microbial/viral contamination (if relevant)	Targeted PCR screening for known pathogens	As needed	-

**Table 5:** Comparative overview of zebrafish embryonic cell lines and live embryo cultures, based on technical, logistical, and ethical parameters.

Aspect	Zebrafish Embryonic Cell Lines	Live Embryo Cultures	Refs.
Initial cost	High due to derivation (enzymatic dissociation, supplements, genotyping)	Low; embryos are externally fertilized and easy to obtain	[9,10,25]
Maintenance cost	Low after establishment (cryopreservation, stable growth)	High due to feeding, water control, animal facilities	[53,54]
Scalability and throughput	High; compatible with HTS in 96-/384-well formats, rapid endpoint assays	Moderate; limited by manual handling, longer assay times	[48,51,52]
Assay duration	Short (24–72 h) for cytotoxicity and mechanistic endpoints	Longer (3–7 days or more) depending on developmental stage	[51,54]
Technical complexity	High during establishment; low for routine use	Low setup, but skilled handling needed for repeated scoring	[25,52,54]
Reproducibility	High; defined genotype, culture conditions, and repeatability	Moderate; subject to environmental and biological variation	[48,54]
Ethical/regulatory profile	Strong alignment with 3Rs; embryos < 5 dpf not regulated as animal models	3Rs compliant, but still considered animal experimentation	[9,54]
Best suited for	High-throughput screening, mechanistic assays, CRISPR validation	Developmental biology, systemic toxicity, regulatory testing	[51,52,54]

## Data Availability

No new data were created or analyzed in this study.

## Abbreviations

The following abbreviations are used in this manuscript:

3Rs	Replacement, Reduction, and Refinement (principios éticos en investigación animal)
ALP	Alkaline Phosphatase
AP	Alkaline Phosphatase
ATAC-seq	Assay for Transposase-Accessible Chromatin using sequencing
ATCC	American Type Culture Collection
bFGF	Basic Fibroblast Growth Factor
CD24	Cluster of Differentiation 24
CMV	Cytomegalovirus (viral promoter)
CRISPR	Clustered Regularly Interspaced Short Palindromic Repeats
CRISPRa/i	CRISPR activation/interference
*ctgfa*	Connective Tissue Growth Factor a
*cyp1a1*	Cytochrome P450 1A1
DMEM	Dulbecco’s Modified Eagle Medium
DMEM/F12	DMEM supplemented with Ham’s F12 Nutrient Mix
DRCF	Danio rerio Caudal Fin cell line
ECACC	European Collection of Authenticated Cell Cultures
ESC	Embryonic Stem Cell
FBS	Fetal Bovine Serum
*fli1*	Friend Leukemia Virus Integration 1
G418	Geneticin (aminoglycoside antibiotic)
*gdf3*	Growth Differentiation Factor 3
GFP	Green Fluorescent Protein
HEPES	4-(2-hydroxyethyl)-1-piperazineethanesulfonic acid (buffering agent)
hESC	Human Embryonic Stem Cell
hpf	Hours post-fertilization
*kdr*	Kinase Insert Domain Receptor (VEGFR2)
*klf4*	Kruppel-like Factor 4
KO	Knockout
L-15	Leibovitz’s L-15 Medium
*lin28*	LIN28 Homolog A
MPC	2-methacryloxyloxyethyl phosphorylcholine
*nanog*	Homeobox protein Nanog
*oct4*	Octamer-binding transcription factor 4 (pou5f1)
PAC2	Zebrafish embryonic fibroblast cell line derived from 24 hpf embryos
PCR	Polymerase Chain Reaction
pEGFP-N1	Plasmid expressing Enhanced Green Fluorescent Protein under CMV promoter
PGC	Primordial Germ Cell
pou5f1	POU domain class 5 transcription factor 1
ronin	Required for Nuclear Factor Activation in Stem Cells
RSV	Rous Sarcoma Virus
RTS34st	Rainbow Trout Spleen Stromal Cell Line
*sall4*	Spalt Like Transcription Factor 4
SDF-1b	Stromal Cell-Derived Factor 1 beta
*sox2*	SRY-box Transcription Factor 2
SSEA	Stage-Specific Embryonic Antigen
STR	Short Tandem Repeat
TCDD	2,3,7,8-Tetrachlorodibenzo-p-dioxin
*tcf3*	Transcription Factor 3
*tdgf1*	Teratocarcinoma-Derived Growth Factor 1
Tol2	Transposon originally from Oryzias latipes
TRA-1-60/81	Tumor-Related Antigens
*vasa*	DEAD-box Helicase 4 (germ cell marker)
Z428	Zebrafish embryonic stem-like cell line
ZBE3	Zebrafish Blastula-derived Embryonic cell line with high transfection efficiency
ZEB2J	Zebrafish Embryonic Blastula-derived cell line adapted from co-culture
ZEM-2 ZEM-2A ZEM2S	Zebrafish Embryonic cell lines with varying serum/media conditions
ZES1	Zebrafish embryonic stem-like cell line
ZF4	Zebrafish Fibroblast cell line
ZFL	Zebrafish Liver cell line
zPDX	Zebrafish Patient-Derived Xenograft
